# Effects of exogenous insulin supplementation on lipid metabolism in peripartum obese dairy cows

**DOI:** 10.3389/fvets.2024.1468779

**Published:** 2025-01-15

**Authors:** Yuanyin Guo, Yihan Zhao, Ziang Wei, Jie Cao

**Affiliations:** College of Veterinary Medicine, China Agricultural University, Beijing, China

**Keywords:** insulin, obese dairy cows, peripartum, subcutaneous adipose tissue, transcriptome

## Abstract

Cows with high body condition scores experience more severe negative energy balance (NEB) and undergo mobilization of more body fat during the peripartum period, leading to more production of nonesterified fatty acids (NEFA) and *β*-hydroxybutyric acid (BHBA). Postpartum insulin secretion is lower, and insulin resistance is stronger in obese cows. Exogenous insulin supplementation has been hypothesized as a key approach for regulating NEFA in these cows. In this study, we assessed the effects of exogenous insulin supplementation on lipid metabolism, key genes regulated by insulin, and the underlying regulatory mechanism. We selected 181 periparturient multiparous obese dairy cows for the study. Cows in the insulin group (*n* = 96) received subcutaneous injections of 200 IU insulin (5 mL) on postpartum days 1 and 7, while cows in the control group (*n* = 85) received subcutaneous injections of 5 mL physiological saline on the same days. The incidence of ketosis was recorded and compared between the two groups. The results demonstrated that postpartum insulin injections significantly reduced the incidence of type II ketosis and delayed the onset time. Meanwhile, a cohort experiment was conducted on 20 cows selected from 181 field trial cows, with 10 cows in the insulin group and 10 cows in the control group. Blood samples were collected for biochemical indicators and subcutaneous adipose tissue was collected for paraffin-embedding and sectioning and RNA sequencing analysis. The results showed that insulin supplementation postpartum reduced concentrations of NEFA and BHBA as well as BCS loss, but did not affect glucose. Additionally, the expression of SREBF1 in insulin signaling pathway and the downstream-regulated lipogenesis network genes were successfully upregulated in insulin-treated healthy group. High expression of SREBF1 may be a key for postpartum insulin supplementation to improve insulin resistance, significantly reduce NEFA concentrations, and prevent or treat ketosis and fatty liver in obese cows. Postpartum administration of insulin could effectively decrease alterations of adipocytes size, which also fully validates that postpartum insulin supplementation promotes lipogenesis and reduces NEFA release.

## 1 Introduction

Dairy cows in the peripartum period experience negative energy balance (NEB). NEB stimulates the mobilization of body fat, leading to the production of nonesterified fatty acids (NEFA) and *β*-hydroxybutyric acid (BHBA). Excessive accumulation of NEFA or BHBA can lead to metabolic diseases such as ketosis and fatty liver (1–3). Cows with high body condition scores, characterized by larger fat reserves and reduced adaptability to lactation, experience more pronounced metabolic changes and exhibit more severe NEB during the peripartum period, leading to greater loss of body condition score (BCS) and higher concentrations of NEFA and BHBA. Consequently, obese cows are more susceptible to lipid metabolism disorder (4–6). Another notable endocrine changes observed in peripartum cows is the reduced secretion of insulin and the development of insulin resistance (7). These cows experience a 50% or greater decline in insulin concentrations and insulin sensitivity (8). Obese cows tend to have lower postpartum insulin concentrations and more severe insulin resistance (9). The decrease in postpartum insulin release and the increase in insulin resistance are significantly correlated with the elevation of NEFA (7). Elevated NEFA disrupt the initial steps of the insulin signaling cascade, exacerbating insulin resistance in peripartum cows. This, in turn, establishes a vicious cycle of metabolic disorders (10, 11).

Both metabolic adaptations and endocrine changes in peripartum obese cows, can lead to increased postpartum NEFA concentrations, which is the main reason for metabolic disorders in peripartum obese cows. Insulin, the hormone theoretically most effective in reducing NEFA concentrations, can inhibit catecholamine-stimulated lipolysis and promote lipogenesis. Adipose tissue, the target organ of insulin, plays a vital role in perinatal metabolic adaptation. During the peripartum period, adipose tissue experiences various transformations, including the downregulation of *de novo* lipogenesis and fatty acid re-esterification, the development of insulin resistance in adipocytes (12–14), and the upregulation of basal and catecholamine-stimulated lipolytic activities (15, 16). Insulin injections have historically been employed for treating ketosis and fatty liver (17, 18). However, the role of insulin in regulating lipid metabolism and improving insulin resistance in peripartum obese dairy cows remains contentious. Therefore, investigating the regulatory mechanisms of insulin on lipid metabolism is of significant importance in cows.

The rapid development of high-throughput sequencing technology has provided a new approach to explore the mechanism underlying lipid metabolism in peripartum obese dairy cows. Transcripts per million (TPM) expression levels can be used for comparing gene expression between individual samples (19). We hypothesized that exogenous insulin supplementation postpartum is helpful to reduce blood NEFA concentrations, and explore further the regulatory mechanism of insulin on lipid metabolism and the key genes of insulin to regulate lipid metabolic changes in obese dairy cows combining transcriptome sequencing technology.

## 2 Results

### 2.1 Impact of postpartum insulin supplementation on the incidence of ketosis

The incidence of Type II ketosis in the insulin group was lower than that in the control group, with subclinical and clinical ketosis incidences reduced by 4.14 and 0.36%, respectively. In contrast, the incidence of Type I ketosis in the insulin group was higher than that in the control group, with subclinical and clinical ketosis incidences increased by 3.92 and 0.64%, respectively. However, the overall postpartum ketosis incidence was essentially similar between the two groups (Table 1).

**Table 1:** Impact of postpartum insulin supplementation on the incidence of ketosis.

Group	Type II ketosis		Type I ketosis	
	Subclinical (%)	Clinical (%)	Subclinical (%)	Clinical (%)
Insulin	22.92%	3.17%	23.92%	4.17%
Control	27.06%	3.53%	20.00%	3.53%

A total of 181 periparturient dairy cows were randomly selected to participate in a postpartum insulin supplementation trial. The treatment group comprised 96 cows, which received subcutaneous injections of 200 IU insulin (5 mL) on postpartum day 1 and postpartum day 7, respectively. The control group consisted of 85 cows, which received subcutaneous injections of 5 mL physiological saline on postpartum day 1 and postpartum day 7 following the same procedure. On postpartum days 5, 10, and 15, *β*-hydroxybutyrate (BHBA) levels at the tail root were measured using a handheld blood ketone meter. BHBA levels ≥1.4 mmol/L were defined as subclinical ketosis, and BHBA levels ≥3.0 mmol/L were defined as clinical ketosis. The incidence of Type II ketosis was calculated based on BHBA measurements on postpartum days 5 and 10, while the incidence of Type I ketosis was determined by the first occurrence of BHBA exceeding 1.4 mmol/L and 3.0 mmol/L on postpartum day 15.

### 2.2 Biochemical indicators

**Figure 1:**
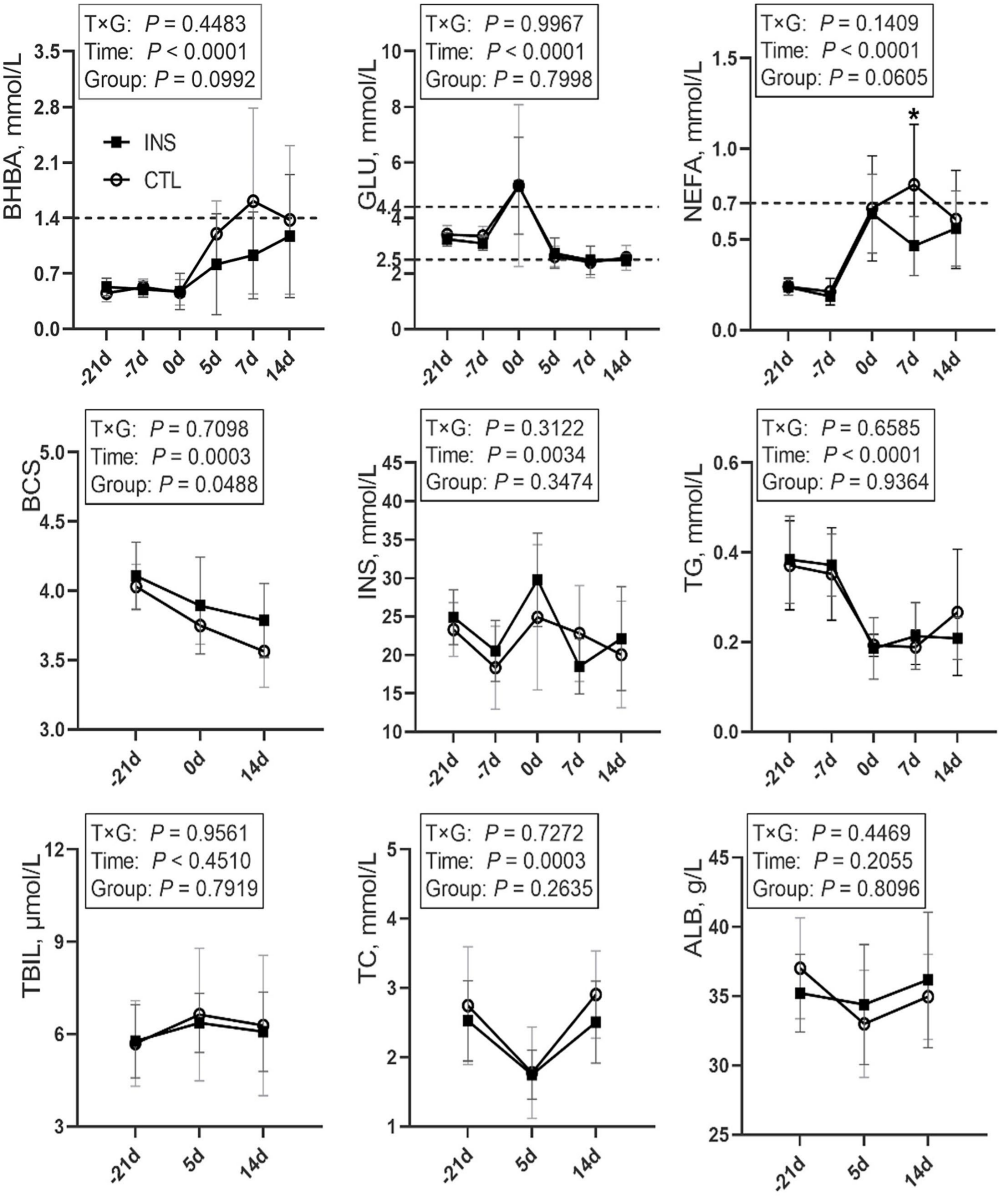
Line chart of blood indices during the perinatal period in the CTL and INS groups. CTL: control group, subcutaneous injection of 5-mL saline on d1 and d7 postpartum; INS: insulin group, subcutaneous injection of 200 U (5 mL) insulin on d1 and d7 postpartum. Error bars indicate the standard error of the mean (SEM); * indicates a significant difference (*p* < 0.05).

Sampling time had significant effects on the blood levels of Glucose (GLU), BHBA, NEFA, and Triglyceride (TG) in obese cows, (*p* < 0.0001) and significant effects on BCS, INS, and TC (*p* < 0.05). No significant differences were observed prepartum between the insulin group and the control group. Assessing the effect of insulin treatment on BHBA and NEFA concentrations and BCS change postpartum, a trend of difference or significant difference (*p* = 0.0992, *p* = 0.0605, *p* = 0.0488) was observed. BHBA in the control group were higher than in the insulin group on day 5, day 7, and day 14 postpartum, and BHBA in the control group on day 7 postpartum was higher than 1.4 mmol/L. BCS was significantly higher in the insulin group than in the control group postpartum. NEFA in the control group were above 0.70 mmol/L on postpartum day 7 and significantly higher than those of the insulin group (*p* = 0.0305). No significant differences were observed in GLU, Insulin (INS), TG, Total cholesterol (TC), Total bilirubin (TBIL), and Albumin (ALB) among groups postpartum (Figure 1).

### 2.3 Adipocyte area

No significant difference was observed in the subcutaneous adipose tissue prepartum (Table 2), which had a significantly larger area than that postpartum (*p* = 0.0024, *p* = 0.0194). The subcutaneous adipose tissue in the insulin group postpartum had a significantly larger area than that in the control group (*p* = 0.0059; Supplementary Figure 1).

**Table 2:** Changes in subcutaneous adipocyte area in peripartum obese cows.

Group	Adipose area
INS_AP	11654.2 ± 2355.7^a^
CTL_AP	11174.6 ± 2273.2^a^
INS_PP	9797.1 ± 2162.5^b^
CTL_PP	8120.1 ± 2377.2^c^

Changes in subcutaneous adipocyte area in peripartum obese cows. Subcutaneous adipose tissue samples were collected on day 21 antepartum and on day 7 postpartum. According to insulin treatment postpartum, the cows were grouped as cows with insulin treatment postpartum (INS_PP), control group postpartum (CTL_PP), insulin group antepartum (INS_AP), and control group antepartum (CTL_AP). Data are presented as mean ± standard error of the mean. Letters a, b, and c denote significant differences (*p* < 0.05).

### 2.4 Differential gene expression and functional enrichment

Principal component analysis (PCA) results revealed the high repeatability and independence among the samples in each group (Supplementary Figure 2), and the samples of the pre- and postpartum groups were completely separated. When comparing the two groups prepartum, only 59 genes had an upregulated expression, and 67 genes had a downregulated expression. Highly overlapping prepartum samples provide a good baseline for postpartum transcriptome analysis.

**Figure 2:**
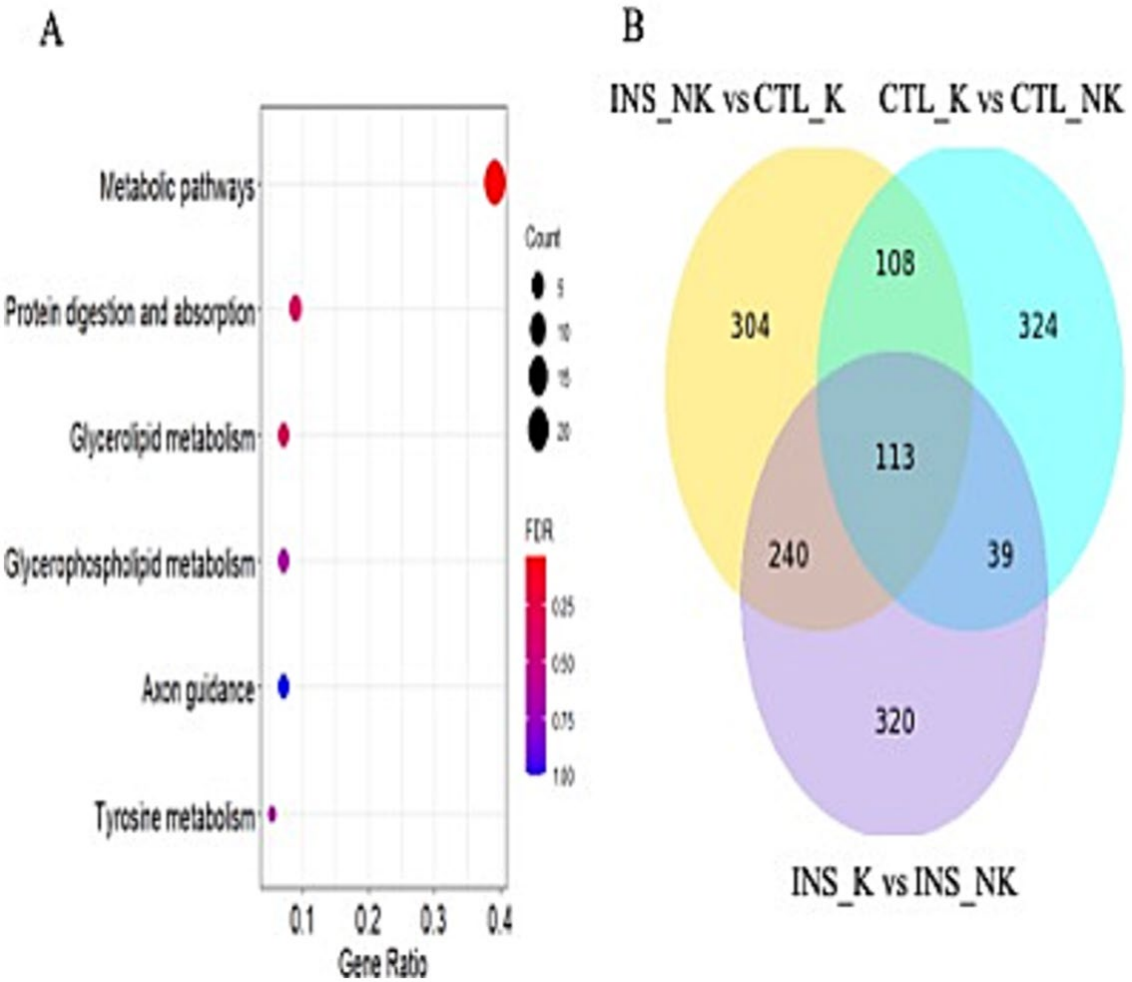
Differentially expressed genes and functional enrichment analysis. **(A)** Volcano plot of the differentially expressed genes between the insulin-treated healthy group (INS_NK) and the control ketosis group (CTL_K). Yellow indicates genes with upregulated expression (log2FC > 1, *p*-value <0.05), blue indicates genes with downregulated expression (log2FC < −1, p-value <0.05); **(B)** KEGG pathway enrichment results of upregulated expression between the INS_NK group and the CTL_K group in peripartum obese cows, (false discovery rate [FDR] < 0.05); **(C)** KEGG enrichment results of common differentially expressed genes in all groups of postpartum obese cows, (FDR < 0.05); **(D)** Venn diagram of common differentially expressed genes of all groups of postpartum obese cows.

Compared with the CTL_K group, 765 differentially expression gene (DEG) were identified in the INS_NK group (Figure 2A). Upregulated genes were significantly enriched in Gene Ontology (GO) terms associated with mitochondria, extracellular space, cell surface, extracellular matrix, cytoplasm, and membrane composition as well as Kyoto Encyclopedia of Genes and Genomes (KEGG) pathways such as tumor necrosis factor (TNF) signaling pathway, mitogen-activated protein kinase signaling pathway, fluid shear stress, atherosclerosis, nuclear factor-κB signaling pathway, adipocyte signaling pathway, AMPK signaling pathway, insulin resistance (MLX interacting protein-like, cyclic AMP-responsive element-binding protein 3-like protein 1, Carnitine palmitoyltransferase 1B, insulin receptor substrate 2, Phosphoenolpyruvate carboxykinase 1, Phosphoenolpyruvate carboxykinase 2, Protein phosphatase 1 regulatory subunit 3C,solute carrier family 2 member 1,solute carrier family 2 member 4, solute carrier family 27 member 6,sterol regulatory element binding transcription factor 1, suppressor of cytokine signaling 3, TNF; *MLXIPL, CREB3L1, CPT1B, IRS2, PCK1, PCK2, PPP1R3C, SLC2A1, SLC2A4, SLC27A6, SREBF1, SOCS3,* and *TNF*), insulin signaling pathway (SHC Adaptor Protein 4,Acetyl CoA carboxylases 1, fatty acid synthase,Hexokinase 2, insulin receptor substrate 2, insulin receptor substrate 3, PCK1, PCK2, cAMP-dependent protein kinase A type II-beta regulatory subunit, PPP1R3C, SLC2A4, SREBF1, suppressor of cytokine signaling 1 and suppressor of cytokine signaling 3; *SHC4, ACACA, FASN, HK2, IRS2, IRS3, PCK1, PCK2, PRKAR2B, PPP1R3C, SLC2A4, SREBF1, SOCS1,* and *SOCS3*; Supplementary Figure 3), African trypanosomiasis, phenylalanine metabolism, cancer pathways, fatty acid metabolism (elongation of very long chain fatty acids protein 6, acetyl acyl coenzyme A-cholesterol acyltransferase 2, ACACA, acyl-CoA synthetase long-chain family member 1, Carnitine palmitoyl transferase IB, FASN, Hydroxysteroid (17b) dehydrogenase type 12, stearoyl-CoA desaturase; *ELOVL6, ACAT2, ACACA, ACSL1, CPTIB, FASN, HSD17B12,* and *SCD*), breast cancer, malaria, hepatocellular carcinoma, type II diabetes (*HK2, IRS2, IRS3, SLC2A, SOCS1, SOCS3,* and *TNF*), glutathione metabolism, metabolic pathways, growth hormone synthesis and secretion, arginine and proline metabolism, and peroxisome proliferator-activated receptor signaling pathway (ACSL1, aquaglyceroporins 7, CPT1B, lipoprotein lipase, Perilipin-2, PCK1, PCK2, SLC27A6, and SCD; *ACSL1, AQP7, CPT1B, LPL, PLIN2, PCK1, PCK2, SLC27A6,* and *SCD*; false discovery rate(FDR) < 0.05; Figure 2B). Downregulated genes were significantly enriched in GO terms associated with membrane composition and extracellular regions as well as KEGG pathways such as complement and coagulation cascades (FDR < 0.05).

Upregulated genes in the CTL_NK group, as compared to the CTL_K group, were enriched in the cytoplasmic vesicle for GO terms and in the RAP1 signaling pathway, estimated glomerular filtration rate tyrosine kinase inhibitor resistance, and PI3K-Akt signaling pathway for KEGG pathway (BCL2-like 1, cyclin-dependent kinase inhibitor 1A, Ephrin A4, fibroblast growth factor receptor 3, the fms-like tyrosine kinase1, kinase insert domain receptor, Lysophosphatidic acid receptor 3, Phosphoinositide-3-kinase regulatory subunit 3, platelet-derived growth factor-like protein, tenascin C, and vascular endothelial growth factor A; *BCL2L1, CDKN1A, EFNA4, FGFR3, FLT1, KDR, LPAR3, PIK3R3, PDGFC, TNC,* and *VEGFA*; FDR < 0.05). Downregulated genes were enriched in GO terms such as extracellular space, extracellular region, extracellular matrix, CCR chemokine receptor binding, ERK1 and ERK2 cascade–positive regulation, chemokine activity, calcium ion binding, IL-1 cellular response, heparin binding, neutrophil chemotaxis, chemokine-mediated signaling pathway, cellular response to tumor necrosis factor, monocyte chemotaxis, extracellular matrix organization, cellular response to interferon-gamma, cell surface, positive regulation of GTPase activity, inflammatory response, skin development, basement membrane, and integrin binding, and no significant enrichment was observed in the KEGG pathway (FDR < 0.05).

Compared with the INS_K group, upregulated genes in the INS_NK group were enriched in GO terms such as extracellular space, transmembrane signaling receptor activity, and GTP binding, with no significant enrichment in the KEGG pathway (FDR < 0.05). Downregulated genes were enriched in GO terms such as extracellular space, with no significant enrichment in the KEGG pathway (FDR < 0.05).

### 2.5 Effect of insulin supplementation on postpartum adipose tissue transcriptome

**Figure 3:**
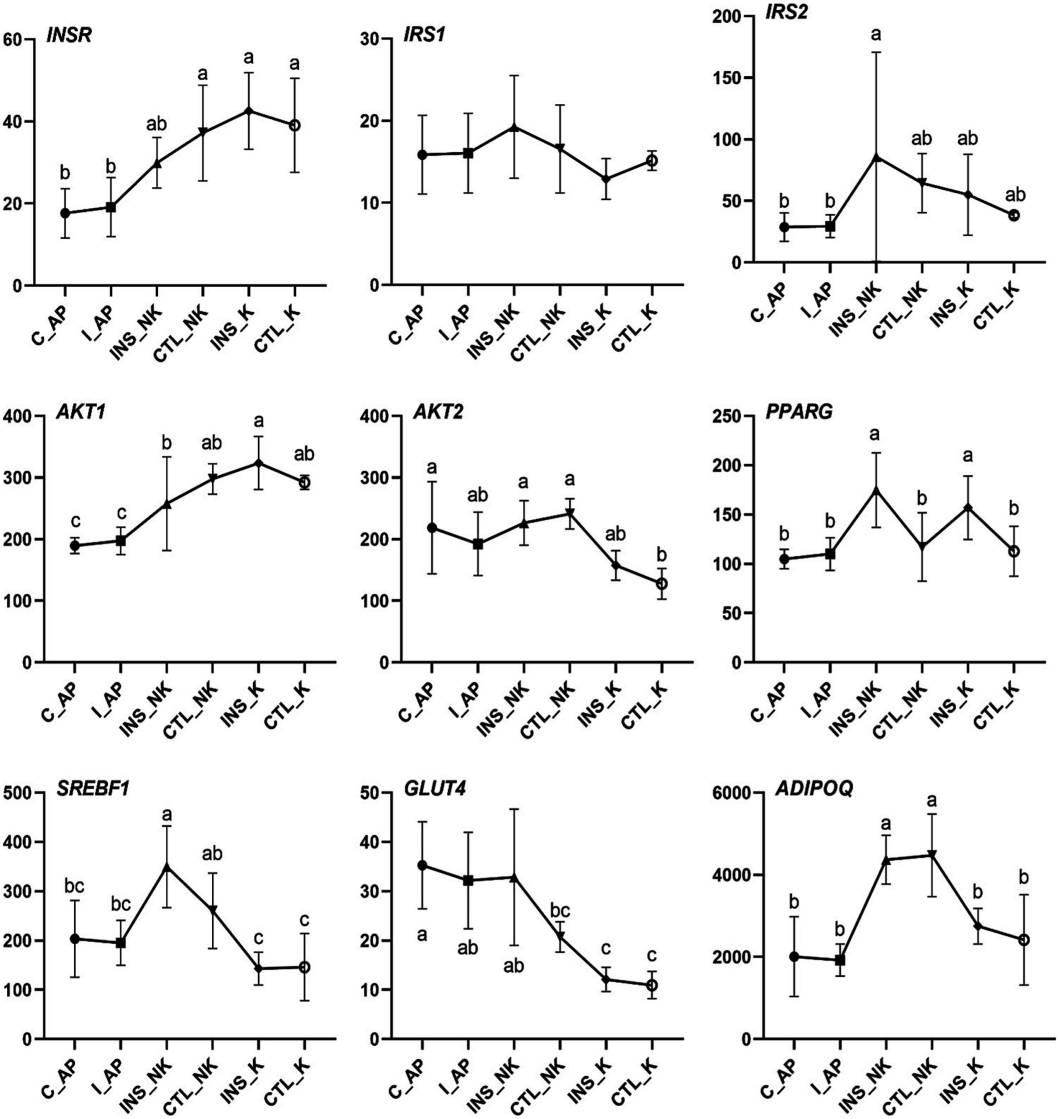
Changes in Transcript per Million (TPM) levels in the insulin signaling pathway in perinatal cows. C_AP: antepartum control group; I_AP: antepartum insulin group; CTL_K: postpartum control ketosis group; CTL_NK: postpartum control healthy group; INS_K: postpartum insulin-treated ketosis group; INS_NK: postpartum insulin-treated healthy group. Error bars indicate the standard error of the mean. Letters a, b, and c denote significant differences (*p* < 0.05).

After Venn analysis of the DEG, 113 DEG were identified (Figure 2D). The common DEG were enriched in GO terms such as extracellular matrix and extracellular space as well as in the KEGG pathway involving metabolic pathways (FASN, glycerol-3-phosphate acyltransferase mitochondrial, SCD, 1-acylglycerol-3-phosphate acyltransferase-2, acyl-CoA synthetase medium chain family members 1, ACSS2, Acyl-CoA thioesterase 2, Adenylosuccinate synthetase1, and diacylglycerol O-acyltransferase 2; *FASN, GPAM, SCD, AGPAT2, ACSM1, ACSS2, ACOT2, ADSS1,* and *DGAT2*; FDR < 0.05; Figure 2C). Based on the enrichment results of common DEG and the physiological characteristics of insulin, the expression of the insulin signaling pathway and the downstream-regulated lipogenic gene network and lipolytic gene network was compared according to log_2_FC and *p*-value (Table 3; Figure 3).

**Table 3:** Comparison of differentially expressed genes involved in the insulin signaling pathway and lipid metabolism.

Gene	INS_NK vs. CTL_K		CTL_K vs. CTL_NK		INS_K vs. INS_NK	
	log_2_FC	*p*-value	log_2_FC	*p*-value	log_2_FC	*p*-value
Insulin signaling pathway						
*INSR*	−0.31	0.33	0.01	0.96	0.42	0.14
*IRS1*	0.39	0.19	−0.17	0.51	−0.65	0.03
*IRS2*	1.30	0.03	−0.79	3.6E-03	−0.79	0.41
*PIK3CA*	−0.02	0.94	0.13	0.57	−0.20	0.48
*AKT1*	−0.09	0.78	−0.10	0.58	0.22	0.47
*AKT2*	0.90	1.3E-03	−0.99	2.7E-06	−0.61	0.02
*PPARG*	0.70	0.03	−0.09	0.75	−0.26	0.36
*SREBF1*	1.32	8.4E-04	−0.89	0.02	−1.39	2.6E-07
*GLUT4*	1.63	5.4E-06	−0.99	4.4E-04	−1.50	7.0E-06
*ADIPOQ*	0.92	0.02	−0.95	0.01	−0.75	1.4E-03
Lipogenic gene network						
*FASN*	3.40	3.3E-11	−1.81	2.0E-05	−3.65	3.3E-11
*ACACA*	1.35	0.01	−0.48	0.21	−1.39	5.5E-04
*SCD*	4.84	3.6E-23	−3.50	2.5E-04	−3.92	2.9E-16
*DGAT1*	0.58	0.09	−0.20	0.51	−0.50	0.04
*DGAT2*	2.41	7.6E-15	−1.91	1.1E-04	−2.37	8.3E-14
*GPAM*	2.57	1.1E-06	−1.28	0.01	−2.50	6.4E-08
*LPL*	2.18	9.9E-12	−2.19	1.0E-13	−2.34	3.1E-13
*ELOVL6*	1.40	1.6E-05	−0.84	2.0E-03	−1.60	6.4E-05
*THRSP*	2.19	2.1E-08	−0.65	0.13	−2.86	3.6E-09
*PCK1*	1.95	1.6E-06	−0.70	0.05	−1.69	2.0E-05
*PCK2*	1.47	1.7E-04	−1.07	2.1E-03	−0.89	1.2E-03
Lipolytic gene network						
*LIPE*	0.29	0.43	−0.37	0.24	−0.24	0.39
*MGLL*	−0.11	0.69	0.37	0.11	0.06	0.80
*PNPLA2*	0.54	0.09	−0.24	0.38	−0.50	0.03

Comparison of differentially expressed genes involved in the insulin signaling pathway and lipid metabolism. Subcutaneous adipose tissue was collected from the tail root area within 2 ~ 4 h after insulin injection on day 7 postpartum. According to insulin treatment and ketosis status, the cows were grouped into insulin-treated ketosis group (INS_K), insulin-treated healthy group (INS_NK), control ketosis group (CTL_K), and control healthy group (CTL_NK). INS_NK vs. CTL_K represents a comparison between INS_NK group and CTL_K group, CTL_K vs. CTL_NK represents a comparison within the control groups, and INS_K vs. INS_NK represents a comparison within the insulin groups.

**Figure 4:**
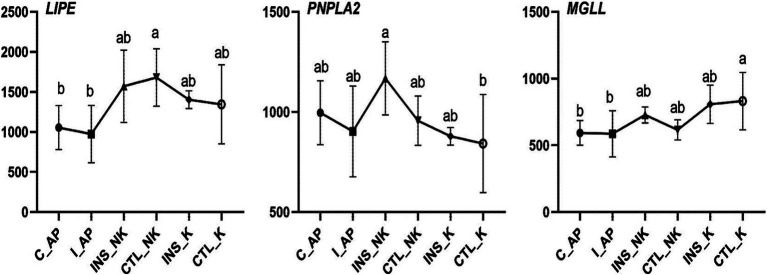
Changes in Transcript per Million (TPM) in the lipolytic gene network in perinatal cows. C_AP: antepartum control group; I_AP: antepartum insulin group; CTL_K: postpartum control ketosis group; CTL_NK: postpartum control healthy group; INS_K: postpartum insulin-treated ketosis group; INS_NK: postpartum insulin-treated healthy group. Error bars indicate the standard error of the mean. Letters a, b, and c denote significant differences (*p* < 0.05).

**Figure 5:**
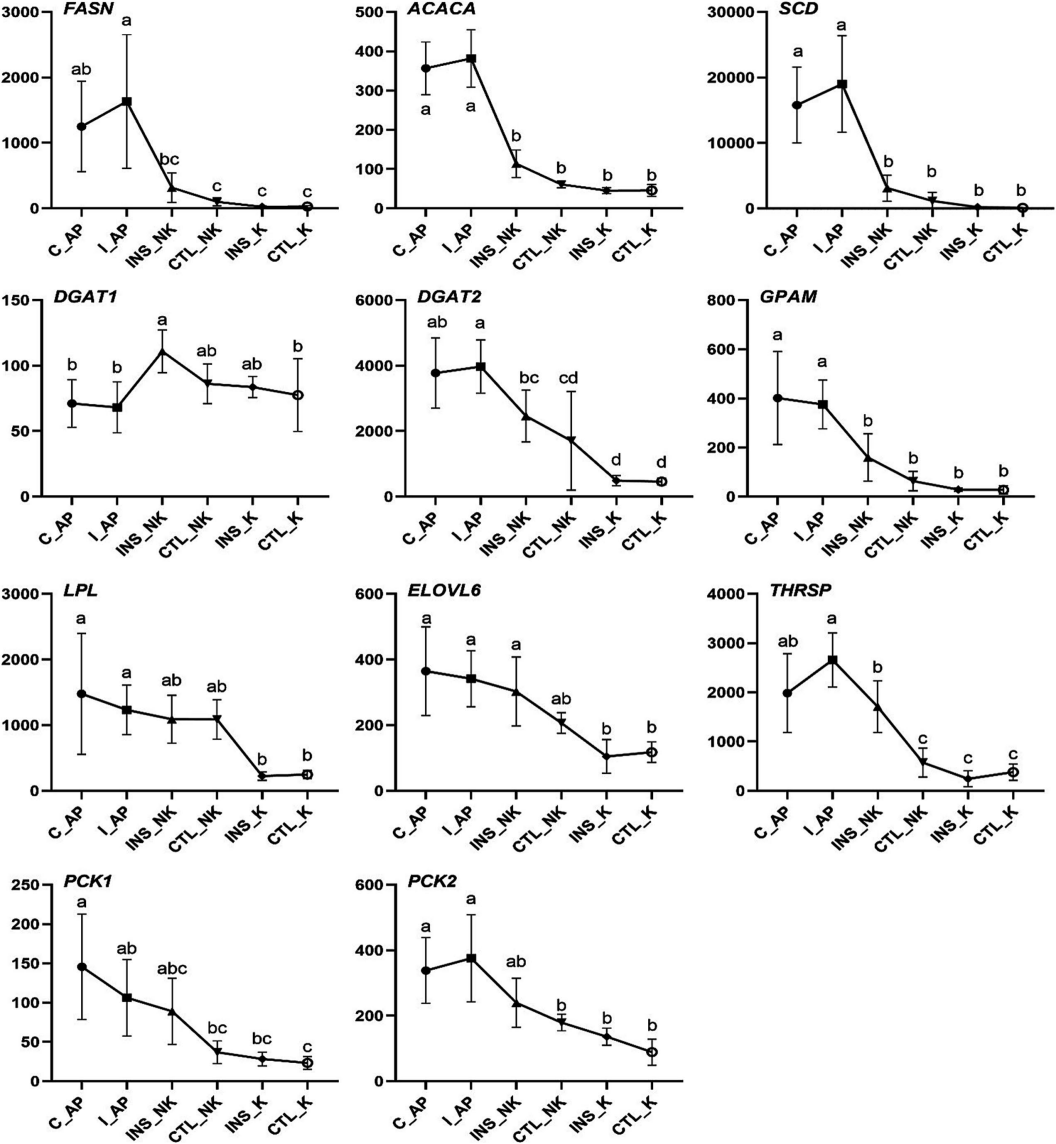
Changes in Transcript per Million (TPM) in the lipogenic gene network in perinatal cows. C_AP: antepartum control group; I_AP: antepartum insulin group; CTL_K: postpartum control ketosis group; CTL_NK: postpartum control healthy group; INS_K: postpartum insulin-treated ketosis group; INS_NK: postpartum insulin-treated healthy group. Error bars indicate the standard error of the mean. Letters a, b, and c denote significant differences (*p* < 0.05).

In the four groups, the expression levels of genes involved in the insulin upstream and downstream signaling pathways, including IRS2, AKT2, SREBF1, and glucose transporter 4 (GLUT4), were significantly lower in the postpartum control ketosis group compared to the control healthy group. Notably, SREBF1 exhibited significantly higher expression in the postpartum healthy group and significantly lower expression in the postpartum ketosis group. The expression of ADIPOQ in the insulin-healthy group was significantly higher than that in both the insulin-ketosis group and the control-ketosis group, while the expression of ADIPOQ in the control-ketosis group was significantly lower than that in the control-healthy group. The expression of PPARG was significantly increased in the comparisons between INS_NK and CTL_NK as well as between CTL_K and INS_K. However, no significant differences were observed in the expression levels of AKT2, PPARG, SREBF1, and GLUT4 between the INS_NK and CTL_NK groups or between the CTL_K and INS_K groups. Under ketosis conditions, insulin supplementation tended to increase the expression of AKT2, PPARG, SREBF1, and GLUT4. Similarly, in the healthy group, insulin supplementation showed a tendency to enhance the expression of SREBF1 and GLUT4 (Figure 4). Additionally, there were no significant differences in the expression of lipid synthesis network genes between the two prepartum groups, and their expression levels were significantly higher than those in the postpartum groups. Postpartum log2FC and Padj results showed that among the lipid synthesis network, adipogenesis-related genes (FASN, ACACA, DGAT1, DGAT2, GPAM, LPL, ELOVL6) and genes regulating adipogenesis (SCD, THRSP, PCK1, PCK2) exhibited no significant differences between the INS_NK and CTL_NK groups or between the CTL_K and INS_K groups. However, these genes were significantly and cooperatively upregulated in the insulin-healthy group, while their expression levels in the ketosis groups were consistently lower than those in the healthy groups (Figure 5).

### 2.6 qRT-PCR assay

**Figure 6:**
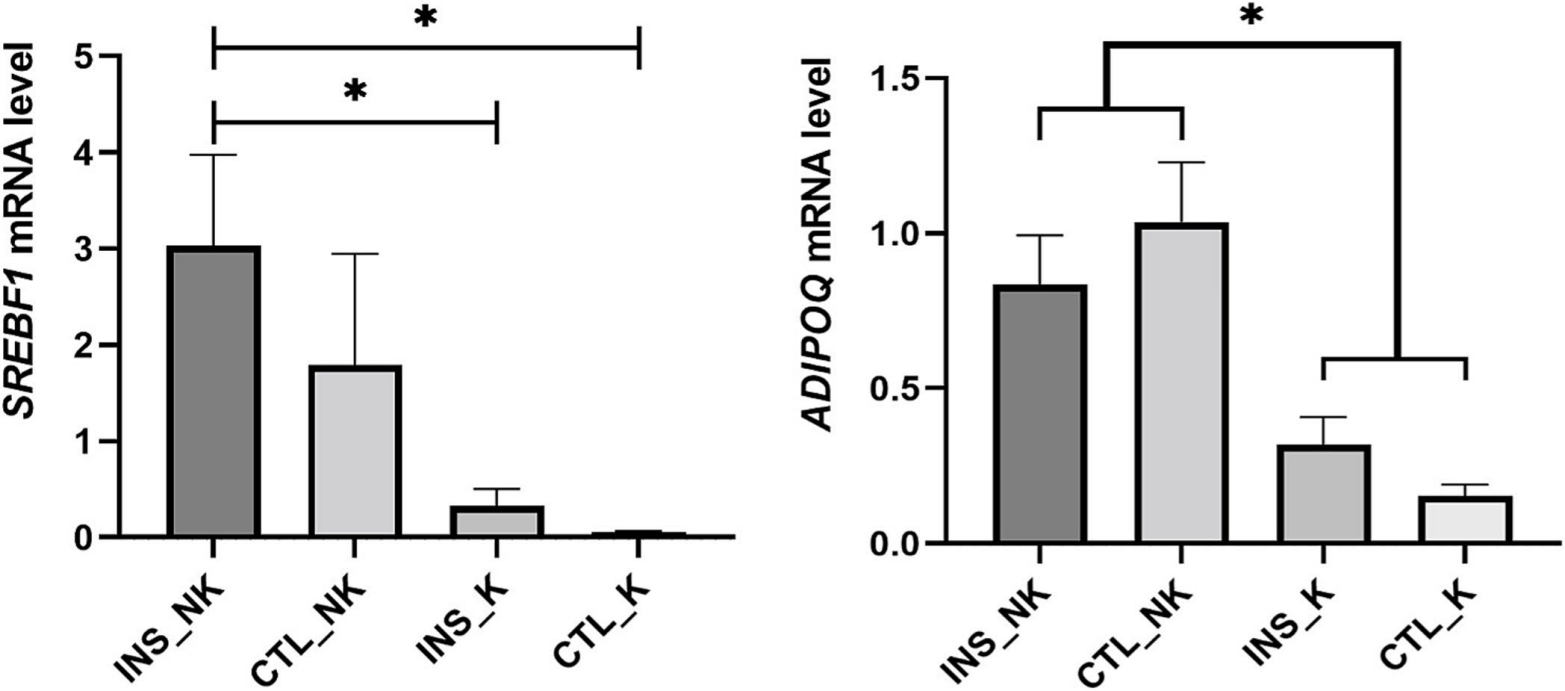
Validation of key candidate genes. Divided into the insulin-treated healthy group (INS_NK), insulin-treated ketosis group (INS_K), control ketosis group (CTL_K), and control healthy group (CTL_NK) according to postpartum insulin treatment and ketosis status. Error bars indicate the standard error of the mean, * indicates significant differences (*p* < 0.05).

Through validation of the expression of key genes (Figure 6), the relative mRNA abundance of *SREBF1* and Adiponectin (*ADIPOQ*) were similar to those in TPM. The mRNA expression of *SREBF1* was highest in the INS_NK group, which was significantly higher than that in the CTL_K group and INS_K group (*p* = 0.0336, *p* = 0.0470). The mRNA expression of *ADIPOQ* was significantly higher in the INS_NK group and the CTL_NK group than in the CTL_K group and the INS_K group (*p* = 0.0138, *p* = 0.0470).

## 3 Discussion

The incidence data of ketosis indicate that postpartum insulin injections can temporarily reduce the incidence of Type II ketosis in obese dairy cows, delay the onset of Type II ketosis, and convert approximately 15% of Type II ketosis cases into Type I ketosis in terms of onset time and severity. Type I ketosis is generally easier to treat.

The severity and duration of peripartum NEB can be determined through the increase in blood NEFA and BHBA and the decrease in the blood GLU (20, 21). Elevated NEFA and BHBA, along with decreased GLU, have been linked to increased risks of ketosis, fatty liver, impaired reproductive ability, and reduced milk yield (22, 23). Postpartum obese cows, often exhibit higher NEFA, elevated NEFA can hinder downstream insulin signaling events and worsen insulin resistance (11, 24). Severe insulin resistance can further cause NEFA to rise, the accumulation of NEFA exacerbates the degree of insulin resistance. This may lead to a vicious cycle of physiological conditions in the body (7, 25). The concentrations of BHBA, NEFA, and GLU, as well as BCS, remained consistent prepartum. However, NEFA in the insulin group on day 7 postpartum was significantly lower than that in the control group, and it consistently remained below 0.70 mmol/L, which indicates a significant reduction in the risk of ketosis and fatty liver in cows (26). By providing exogenous insulin supplementation postpartum, NEFA in obese cows, can be effectively lowered below the 0.70 mmol/L threshold. Similarly, BHBA in the insulin group was lower than that in the control group on days 5, 7, and 14 postpartum, remaining below the diagnostic threshold of 1.4 mmol/L for subclinical ketosis (27–29). Exogenous insulin supplementation postpartum can effectively regulate BHBA within the normal range in obese cows. BCS loss is positively correlated with NEFA concentrations in lactating cows. Avoiding severe BCS loss during the peripartum period is significant for preventing an imbalance in lipid metabolism (30). BCS in the insulin group was significantly higher than that in the control group. Exogenous insulin supplementation postpartum can effectively prevent excessive BCS loss in obese cows, during the peripartum period. Exogenous insulin supplementation postpartum did not have a significant impact on GLU and INS. Insulin resistance is more pronounced in cows with higher body condition scores (9). In our study, the experimental cows had high body condition scores (3.75–4.25), and insulin injection did not reduce blood glucose levels. Instead, insulin likely primarily influenced glucose utilization, as indicated by increased GLUT4 expression. From the perspective of energy metabolism, maintaining blood glucose levels represents a positive effect of insulin in controlling ketosis. To summarize, the subcutaneous administration of 200 U of insulin on days 1 and 7 postpartum does not decrease GLU, while can significantly reduce postpartum BHBA and NEFA in obese cows, as well as mitigate the loss of BCS during the peripartum period, which are crucial for the prevention and treatment of metabolic disorders.

The concentration of NEFA is influenced by the balance between lipolysis and lipogenesis in adipose tissue (31). The size of individual adipocytes is mainly defined by LD size, as adipocytes are composed of more than 95% triglycerides (TG), which are hydrolyzed into glycerol and fatty acids (FAs) during the process of fat breakdown (32). Triglyceride lipase, as the main rate-limiting enzyme in adipocyte lipolysis, also determines the size of LD (33). A decrease in adipocyte size indicates the release of TG from adipocytes (34, 35). Smaller adipocytes postpartum indicate a higher breakdown of TG into NEFA, which are released into the blood and liver. This overall decrease in adipocyte size is consistent with the metabolic shift from an anabolic to a catabolic state in peripartum cows (16, 36, 37). The excessive release of NEFA from adipose tissue is the primary factor contributing to severe metabolic issues and an increased risk of ketosis and fatty liver in peripartum cows. The metabolic adaptation of adipose tissue is crucial for a smooth transition during the peripartum period (38, 39). Postpartum BCS and subcutaneous adipocyte area in dairy cows were significantly lower compared to prepartum levels. Additionally, there was no significant difference in adipocyte area prepartum, but the insulin group exhibited significantly larger adipocyte area compared to the control group postpartum. Exogenous insulin supplementation postpartum effectively reduced the change in adipocyte size. It achieved dynamic balance in the decomposition and remodeling of adipose tissue, thereby decreasing TG breakdown, NEFA release in adipocytes, and mitigating intense lipolysis caused by more severe NEB in obese cows.

The expression of the lipogenic genes did not show significant differences prepartum and was significantly higher than that in postpartum cows in our study. The expression of lipogenic genes postpartum displayed coordinated downregulation in subcutaneous white adipose tissue of cows (40, 41). The downregulated expression of lipogenic genes is closely associated with the reduced rate of lipogenesis, because lipogenesis is primarily controlled by gene expression at the transcriptional level (42). The mRNA expression of genes that are lipogenic enzymes such as *FASN, ACACA, DGAT1, DGAT2, GPAM, LPL,* and *ELOVL6*, as well as genes that regulate lipogenesis like *SCD, THRSP, PCK1,* and *PCK2*, displayed significant upregulated expression in the INS_NK group. In particular, the genes *FASN* (log_2_FC = 3.40), *ACACA* (log_2_FC = 1.35), *SCD* (log_2_FC = 4.84), *DGAT1* (log_2_FC = 0.58), *DGAT2* (log_2_FC = 2.41), and *LPL* (log_2_FC = 2.18) exhibited notable fold changes in the INS_NK group. Their expression levels in the ketosis group were significantly lower compared to the healthy group. The lipogenic genes were actived successfully by exogenous insulin supplementation postpartum in adipose tissue, which may mainly explain the decrease in blood NEFA in obese cows.

In early lactating cows, the mRNA expression of lipolytic enzymes in the adipose tissue is upregulated moderately and further increases as milk production increases (40, 41, 43). The expression of key lipolytic enzymes (lipase, patatin-like phospholipase domain-containing protein 2, and monoglyceride lipase; *LIPE, PNPLA2,* and *MGLL*) is similar to changes in the expression pattern of genes regulating lipogenesis, and both decrease after calving (36). In our study, no significant differences were observed postpartum in the expression of lipolysis-related genes like *LIPE, PNPLA2,* and *MGLL*. Lipolysis in the early lactation is primarily regulated by the degree of the post-translational phosphorylation activity rather than the expression of key lipolytic enzymes at the transcriptional level (44–46). Thus, the importance of lipolysis dysregulation postpartum in the formation and development of ketosis and fatty liver cannot be ignored. Insulin inhibits protein kinase A activation on hormone-sensitive triglyceride lipase (HSL) phosphorylation sites by reducing the cyclic AMP concentration (47, 48). In-depth studies at the protein level are still needed to further understand the effect of insulin on lipolysis in peripartum obese cows. Previous studies have shown that insulin can promote lipid synthesis and inhibit lipolysis (49). However, our experimental results indicate that exogenous insulin supplementation has no effect on lipolysis. This may be attributed to the high body condition scores (3.75–4.25) of the cows used in our study. In postpartum dairy cows experiencing negative energy balance, severe body fat mobilization is commonly observed, which likely masked the inhibitory effect of exogenous insulin on lipolysis.

Both lipolysis and lipogenesis in adipose tissue are regulated by circulating insulin concentrations and the responsiveness and sensitivity to insulin (7). Compared with the CTL_K group, upregulated genes of the INS_NK group were significantly enriched in insulin signaling pathway (FDR < 0.05), and were concentrated in the downstream pathway promoting the lipogenesis effects. Postpartum dairy cows have lower insulin concentrations and higher insulin resistance (50). Prepartum obesity makes cows more susceptible to insulin resistance postpartum, increasing the risk of developing metabolic disorders (9). Reduced mRNA expression of *AKT2*, *SREBF1* and *GLUT4* in the subcutaneous adipose tissue, as well as the downregulation of *SREBF1*-regulated lipogenic genes, indicated insulin resistance occurred in early lactation cows (43). Except for PPARG, which showed significant differences between the INS_NK and CTL_NK groups as well as between the CTL_K and INS_K groups, no significant differences were observed for other genes in these comparisons. However, under ketosis conditions, insulin supplementation tended to increase the expression of AKT2, PPARG, SREBF1, and GLUT4. Similarly, in the healthy group, insulin supplementation also exhibited a tendency to enhance the expression of PPARG, SREBF1, and GLUT4. These findings suggest that postpartum insulin supplementation in dairy cows may partially improve insulin resistance. However, the gene expression of *AKT2, SREBF1,* and *GLUT4,* and lipogenic genes regulated by insulin was significantly lower in the INS_K group, indicating that some cows have more severe insulin resistance that cannot be relieved by exogenous insulin supplementation. In the CTL-K group, the gene expression of the insulin signaling pathway, namely, *IRS2, AKT2, SREBF1,* and *GLUT4*, was significantly lower than that in the CTL_NK group, consistent with the findings of previous research suggesting the stronger insulin resistance in ketosis cows (51, 52).

Insulin primarily exerts its effects through the activation of the transcription factor *SREBF1* (49), which is mainly regulated by insulin (53). *SREBF1* encodes the sterol regulatory element-binding protein 1, activating the expression of lipogenic genes including *FASN, ACACA, SCD, GPAM, DGAT1,* and *DGAT2* (54, 55). Consequently, it promotes the synthesis of palmitic acid, oleic acid, and TG, while reducing the concentration of NEFA in the blood and liver. *SREBF1* expression was highest in the INS_NK group, followed by the CTL_NK group, both of which exhibited significantly higher expression compared to the ketosis groups. The increased mRNA expression of *SREBF1* induced by insulin supplementation in obese cows, is a critical factor for significantly upregulating lipogenic genes, and also a key target for improving insulin resistance and the significant reduction in NEFA in obese cows. Furthermore, *SREBF1* expression remained higher in CTL_NK group even without insulin supplementation, indicating that some cows exhibited lower insulin resistance, contributing to reduced NEFA and a smoother metabolic adaptation in obese cows. *ADIPOQ* encodes adiponectin (ADPN), an insulin sensitizer that promotes lipogenesis and inhibits lipolysis (56, 57). ADPN concentrations in the circulatory system and various fat depots of high-yielding cows decrease during the peripartum period, reaching their lowest point immediately after calving (58, 59). Additionally, *ADIPOQ* mRNA expression in subcutaneous adipose tissue is downregulated (60). Postpartum ADPN concentrations are associated with insulin resistance and are sensitive to energy balance in peripartum cows. Higher postpartum *ADIPOQ* expression in cows helps balance lipogenesis and lipolysis. Among the groups, the highest postpartum *ADIPOQ* expression was observed in the CTL_NK group, followed by the INS_NK group, with both groups showing significantly higher expression compared to the postpartum ketosis cows. The elevated expression of *ADIPOQ* in the CTL_NK group may explain their higher S*REBF1* expression and improved insulin resistance. Conversely, the low expression of *ADIPOQ* in the INS_K group could contribute to their lower *SREBF1* expression and more severe insulin resistance.

## 4 Conclusion

Administering a subcutaneous injection of 200 U of insulin on days 1 and 7 postpartum in Cows with high body condition scores, effectively reduces BHBA, NEFA and BCS loss, without impacting GLU. This intervention proves beneficial for regulating metabolic disorders in peripartum obese cows. Postpartum insulin supplementation successfully activates the insulin signaling pathway and the downstream-regulated lipogenesis gene network, improving insulin resistance, which may explain the significant decrease in blood NEFA levels we observed. Notably, the high expression of *SREBF1* postpartum induced by insulin supplementation may serve as a key point to relieve insulin resistance, lower NEFA concentrations in obese cows. Insulin promotes lipogenesis and reduces NEFA release, which has been fully confirmed in adipocyte area changes. Postpartum insulin supplementation significantly reduces the change of adipocyte size in obese cows, which is beneficial to buffer the intense lipolysis and adipose tissue remodeling caused by more severe NEB during the peripartum period.

## 5 Materials and methods

### 5.1 Animals

The longitudinal cohort study was conducted in a large-scale intensive farm (Hebei Province, China). A total of 181 periparturient dairy cows, with parities ranging from 2 to 5 and body condition scores (BCS) between 3.75 and 4.25, were randomly selected to participate in a postpartum insulin supplementation trial. The treatment group comprised 96 cows, which received subcutaneous injections of 200 IU insulin (5 mL) on postpartum day 1 and postpartum day 7, respectively. Insulin was purchased from Jiangsu Wanbang (400 U/10 mL; H10890001, Becton Dickinson and Company). When subcutaneously administered, insulin was quickly absorbed and began to show its effect at 0.5 ~ 1 h, reaching the peak effect at 2 ~ 4 h and being maintained at 5 ~ 7 h, with a half-life of 2 h. The control group consisted of 85 cows, which received subcutaneous injections of 5 mL physiological saline on postpartum day 1 and postpartum day 7 following the same procedure. On postpartum days 5, 10, and 15, *β*-hydroxybutyrate (BHBA) levels at the tail root were measured using a handheld blood ketone meter. BHBA levels ≥1.4 mmol/L were defined as subclinical ketosis, and BHBA levels ≥3.0 mmol/L were defined as clinical ketosis. The incidence of Type II ketosis was calculated based on BHBA measurements on postpartum days 5 and 10, while the incidence of Type I ketosis was determined by the first occurrence of BHBA exceeding 1.4 mmol/L and 3.0 mmol/L on postpartum day 15. Cows diagnosed with clinical ketosis received veterinary treatment with glucose and propylene glycol. A total of 20 multiparous obese dairy cows were randomly selected from 181 field trial cows to participate in a longitudinal cohort trial. According to the principle of similar parity and BCS, the cows were divided into two groups based on the parity order of calving (odd or even): the control group (10 cows) and the insulin group (10 cows). The insulin group received insulin treatment, while the control group received physiological saline as a placebo, following the procedures described above. In the cohort trial involving 20 obese dairy cows, 5 cows were excluded due to the development of diseases unrelated to ketosis within 14 days postpartum (including birth canal injury, endometritis, metritis, and retained placenta). As a result, the final sample included 7 cows in the insulin group and 8 cows in the control group. On postpartum day 7, blood samples were collected from the tail vein of each cow, and *β*-hydroxybutyrate (BHBA) levels were measured using a handheld blood ketone meter (FreeStyle Optium Neo H; Abbott Diabetes Care Lid, United Kingdom). Based on the BHBA results on postpartum day 7, the insulin group and the control group were subdivided into the following groups: insulin-treated healthy group (INS_NK), which included cows with BHBA <1.4 mmol/L; insulin-treated ketosis group (INS_K), which included cows with BHBA ≥1.4 mmol/L; control healthy group (CTL_NK), which included cows with BHBA <1.4 mmol/L; and control ketosis group (CTL_K), which included cows with BHBA ≥1.4 mmol/L. The grouping results were used for the analysis of ketosis related insulin, biochemical indicators, and physical condition scores.

### 5.2 Management, sampling, and analyses

During the experiment, the cows fed on fresh total mixed ration (TMR; Table 4) three times a day at 9:30, 15:00, and 20:00, and they had access to water ad libitum for 24 h. Blood samples were collected from the coccygeal vein at 9:00 a.m. before TMR feeding, using a vacuum coagulation tube (BD Vacutainer, United States) and a sodium heparin anticoagulation tube (BD Vacutainer, USA), respectively, on day 7 and day 21 prepartum and on day 0, day 5, day 7, and day 14 postpartum. Subcutaneous adipose tissue was collected within 2–4 h after insulin injection, and blood samples were simultaneously collected from the coccygeal vein to measure glucose and other indicators. Blood samples were placed immediately into an ice box and transported to the laboratory, and then were centrifuged at 3000 rpm for 5 min. 1 mL of serum and 1 mL of plasma were transferred to cryotubes and stored in a − 20°C freezer for further analysis. BHBA and glucose (GLU) were measured immediately after collecting the whole-blood samples from the tail vein. NEFA, insulin (INS), and triglycerides (TG) were measured in the serum samples, and total bilirubin (TBIL), albumin (ALB), and total cholesterol (TC) were measured in the plasma samples. Biochemical assay kits were purchased from Nanjing Jiancheng Bioengineering Institute (China), and DG5033A enzyme-linked immunosorbent assay (ELISA) reader from Huadong Electronics Group Medical Equipment Co., Ltd., was used to perform ELISA. Among the 20 experimental cows, five cows with any postpartum clinical diseases other than ketosis were excluded within 14 days postpartum, and the samples were obtained from seven cows in the insulin group and eight cows in the control group (Supplementary Table 1).

**Table 4:** TMR (Unit: g).

Molasses	Fat powder	DDGS	Pelleted Corn	Calcium fatty acids	Premix	Cottonseed	Soybean meal	Domestic alfalfa	Corn bran powder	Beet pellets
218	150	700	3,600	300	1,100	2000	2,386	967	600	200
Silage	Imported Alfalfa	Wheat Bran	Extruded Soybeans	Corn	Rumen probiotics	Domestic Oat Grass	Soybean Hulls	Alfalfa Silage	Water	Total
25,000	1,000	200	543	6,366	400	1,000	200	2,700	5,600	55,230

### 5.3 Adipose tissue biopsy and adipocyte area calculation

Biopsy samples of adipose tissue were collected from the tail-head region on day 21 prepartum and day 7 postpartum within 2 to 4 h after the second insulin subcutaneous injection, following the method described by Ning et al. (61). One part of the collected adipose tissue was cut into small pieces (about 0.5 cm in diameter) and fixed in RNAlater® and stored in a − 20°C freezer for RNA extraction and further analysis, and the other part was also cut into small pieces (about 0.7 cm in diameter) and fixed using 4% formaldehyde solution and stored at room temperature for preparing paraffin-embedded sections. After the formaldehyde-fixed adipose tissue was sectioned, the adipocyte area was calculated according to the method described by Parlee et al. (62). Adipocyte area was calculated using NDP.view2 software (U12388–01, Hamamatsu, Japan).

### 5.4 RNA-seq analysis

In accordance with the sample size criteria for DEG analysis and considering budget constraints, 12 cows were selected from 15 cows without postpartum related diseases for the experiment, with 3 cows in each group: INS-NK group, INS_K group, CTL-NK group, and CTL_K group. Based on the results of 5 blood sample tests at 0, 5, 7, 10, and 14 days postpartum, any test with BHBA <1.4 mmol/L was classified as the ketosis group (K group), otherwise it is classified as the non-ketosis group (NK group). Total RNA was extracted according to the method described by Ning et al. (63). High-throughput sequencing was performed on qualified RNA samples, and raw sequencing data were subjected to quality control analysis. The results of sequencing and quality control analyses are shown in Supplementary Table 2. The clean data were then aligned, quantified, and analyzed for differential gene expression using STAR v.2.7.5b, RSEM v1.3.2, and DESeq2 v1.32.0, respectively. DEG were defined as those with |Log_2_FC| > 1 and *p*-value <0.05. Functional enrichment analysis of DEG was performed using the DAVID website (DAVID v6.8).^1^

### 5.5 Validation of key candidate genes

The PrimeScript™ RT Reagent Kit with a DNA eraser (Perfect Real Time, TaKaRa, Dalian, China) was used to synthesize cDNA from the extracted RNA according to the manufacturer’s instructions. Quantitative real-time reverse transcription–polymerase chain reaction (qRT-PCR) assay was performed using LightCycler® 480 SYBR-Green I Master (Roche Applied Science, Indianapolis, IN, United States) and CFX Maestro software 1.1 (ver.4.1.2433.1219; Bio-Rad, Hercules, CA, USA). The cycling conditions were as follows: 95°C for 5 min, 39 cycles at 95°C for 10 s, 60°C for 20 s, and 72°C for 20 s. The key genes *SREBF1* and *ADIPOQ* were validated using primers according to the method described by Ji et al. (43). According to Bonnet et al. (64), *UXT*, *EIF3K*, and *TBP* were the most stably expressed genes in bovine adipose tissue. These genes were selected as reference genes, and primer descriptions are provided in Bonnet et al. (64). The relative mRNA abundance of *SREBF1* and *ADIPOQ* was calculated by normalizing the results to the geometric mean of the reference genes. Three technical replicates were used for each biological sample in the experiment.

### 5.6 Statistical analysis

Biochemical results, TPM, and qRT-PCR data were analyzed by GraphPad Prism v9.3.0 (GRAPH PAD Software Inc., San Diego, California, United States) and IBM SPSS 26.0 software (IBM Corp., Armonk, NY, United States). Two-way analysis of variance (ANOVA) was conducted on the biochemical results, while one-way ANOVA was performed on the TPM and qRT-PCR data. The two-way ANOVA model incorporated fixed effects of insulin treatment, time, and the interaction between insulin treatment and time. *p* < 0.05 indicated statistically significant differences.

## Data Availability

The raw data supporting the conclusions of this article will be made available by the authors, without undue reservation.

## Footnotes

^1^ https://david.ncifcrf.gov/
