# Supplementary material for: Effects of exogenous insulin supplementation on lipid metabolism in peripartum obese dairy cows
Source: Front Vet Sci. 2025 Jan 15;11:1468779. doi: 10.3389/fvets.2024.1468779 (PMC11774932; doi:10.3389/fvets.2024.1468779)
Supplement: Supplementary file 1 [file Table_1.DOCX]

Supplementary Material

# Supplementary Tables

**Supplementary Table 1.** Lactation number, BCS, and BHBA in the INS group and the CTL group in the perinatal period.

| Group | Lactation number | BCS (-21d) | BHBA (-21d) | BHBA (7d) |
| --- | --- | --- | --- | --- |
| INS (7) | 3 | 4 | 0.5 | 1.7 |
|  | 2 | 4 | 0.7 | 0.4 |
|  | 3 | 4.5 | 0.6 | 1.4 |
|  | 4 | 4.25 | 0.4 | 0.5 |
|  | 4 | 4 | 0.6 | 1.4 |
|  | 3 | 3.75 | 0.5 | 0.5 |
|  | 5 | 4.25 | 0.4 | 0.6 |
| CTL (8) | 5 | 3.75 | 0.6 | 0.4 |
|  | 2 | 4 | 0.5 | 0.6 |
|  | 3 | 4.25 | 0.3 | 0.5 |
|  | 4 | 4.25 | 0.6 | 1.8 |
|  | 3 | 4 | 0.4 | 1.7 |
|  | 4 | 4 | 0.4 | 3.7 |
|  | 2 | 4 | 0.4 | 2.8 |
|  | 3 | 4 | 0.4 | 1.4 |

CTL: control group, subcutaneous injection of 5-mL saline on d1 and d7 postpartum; INS: insulin group, subcutaneous injection of 200 U (5 mL) insulin on d1 and d7 postpartum.

**Supplementary Table 2.** Statistics for RNA-Seq reads from adipose tissue, and alignment information (group means).

| Items^1^ | -21d | | 7 d | | | |
| --- | --- | --- | --- | --- | --- | --- |
|  | I_AP | C_AP | INS_NK | INS_K | CTL_NK | CTL_K |
| Raw reads | 40968934 | 41909997 | 42486640 | 46716953 | 43529294 | 44115444 |
| Clean reads | 39839056 | 40439342 | 41224757 | 45041393 | 41834075 | 42843065 |
| Raw base (G) | 6.15 | 6.29 | 6.37 | 7.01 | 6.53 | 6.62 |
| Clean base (G) | 5.98 | 6.07 | 6.18 | 6.75 | 6.28 | 6.43 |
| Effective (%) | 97.26 | 96.47 | 97.02 | 96.39 | 96.15 | 97.12 |
| Error (%) | 0.03 | 0.03 | 0.03 | 0.03 | 0.03 | 0.03 |
| Q20 (%) | 97.47 | 97.53 | 97.43 | 97.59 | 97.55 | 97.50 |
| Q30 (%) | 93.34 | 93.48 | 93.31 | 93.60 | 93.53 | 93.42 |
| GC (%) | 51.25 | 51.66 | 51.70 | 51.31 | 51.68 | 51.22 |
| Uniquely mapped (%) | 95.27% | 94.93% | 94.55% | 94.70% | 94.65% | 95.11% |

C_AP: antepartum control group; I_AP: antepartum insulin group; CTL_K: postpartum control ketosis group; CTL_NK: postpartum control healthy group; INS_K: postpartum insulin-treated ketosis group; INS_NK: postpartum insulin-treated healthy group.

# Supplementary Figures


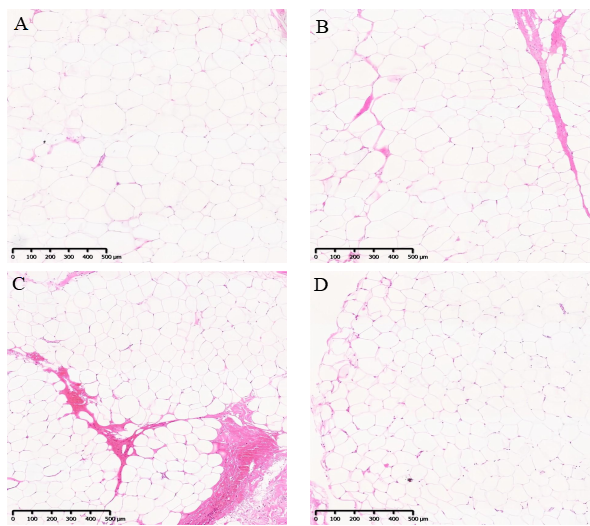


**Supplementary Figure 1.** Changes of the area of perinatal subcutaneous adipose cells in the CTL and INS groups. Subcutaneous adipose tissue samples were collected on d21 antepartum and on d7 postpartum. According to insulin treatment postpartum, the cows were grouped as cows with insulin treatment postpartum (INS_PP), control group postpartum (CTL_PP), insulin group antepartum (INS_AP), and control group antepartum (CTL_AP). A: CTL_AP, B: INS_AP, C: INS_PP, D: CTL_PP.


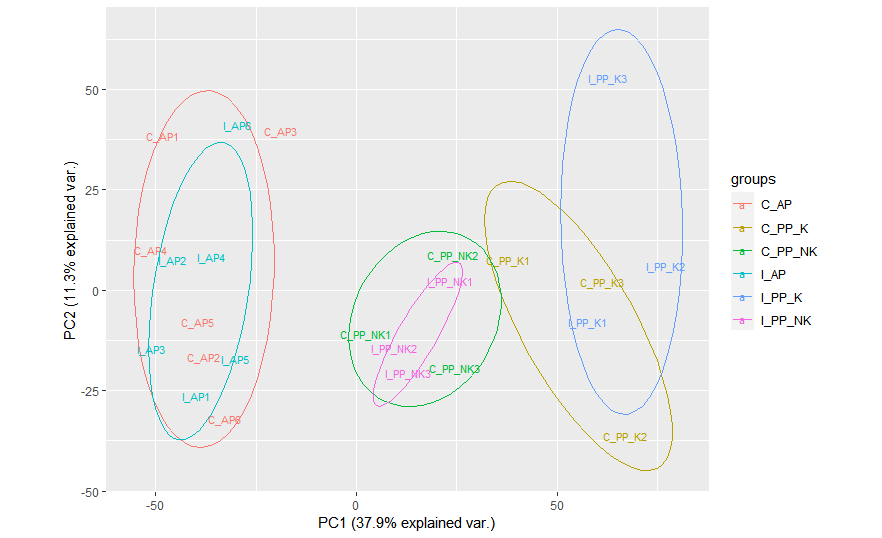


**Supplementary Figure 2.** Principal component analysis of the perinatal transcriptome of the INS and CTL groups. Subcutaneous adipose tissue was collected from the tail root area within 2 ~ 4 hours after insulin injection on d7 postpartum. C_AP: antepartum control group; I_AP: antepartum insulin group; CTL_PP_K: postpartum control ketosis group; CTL_PP_NK: postpartum control healthy group; INS_PP_K: postpartum insulin-treated ketosis group; INS_PP_NK: postpartum insulin-treated healthy group.


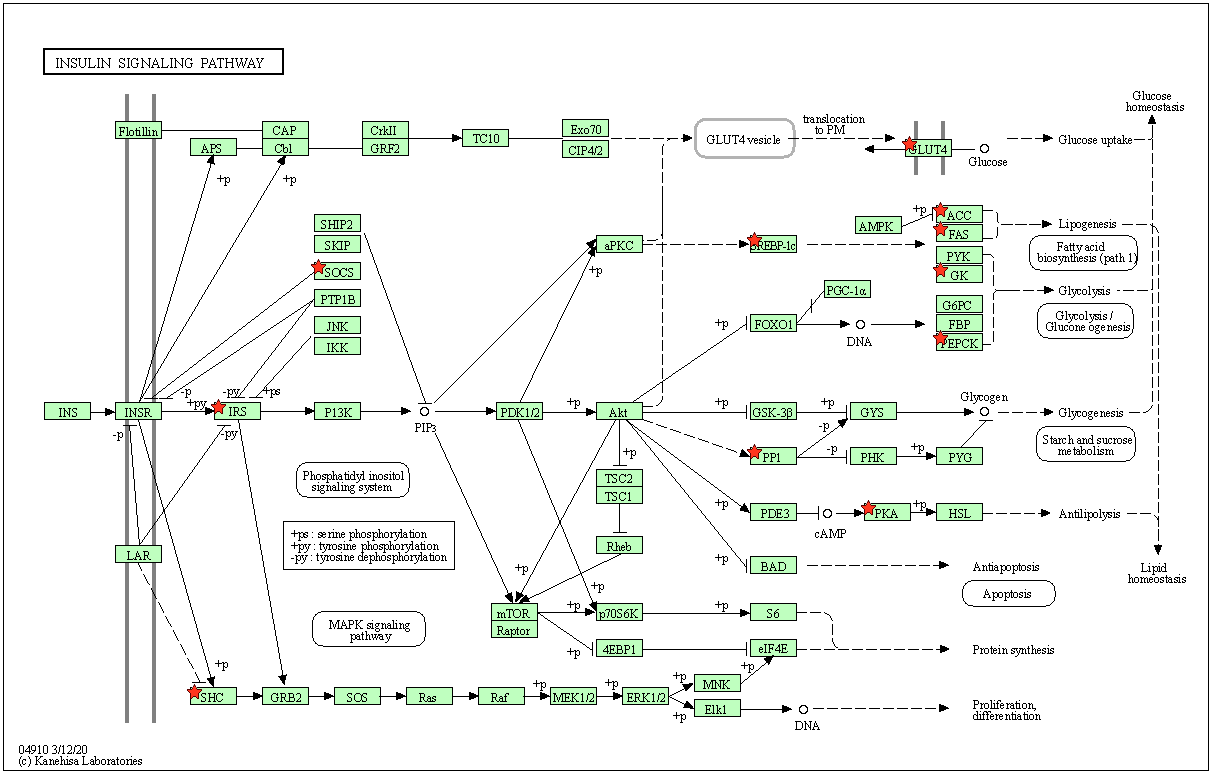


**Supplementary Figure 3.** Insulin signaling pathway. Red asterisks indicate up-regulated genes between the insulin healthy group and the control ketosis group.
